# A bowhead whale in the eastern North Pacific

**DOI:** 10.1002/ece3.8664

**Published:** 2022-03-07

**Authors:** Jared R. Towers, James F. Pilkington, Ernest A. Mason, Ernest V. Mason

**Affiliations:** ^1^ 111142 Bay Cetology Alert Bay British Columbia Canada; ^2^ Fisheries and Oceans Canada Pacific Biological Station Nanaimo British Columbia Canada; ^3^ Kitasoo/Xai'xais Nation Klemtu British Columbia Canada

**Keywords:** *Balaena mysticetus*, bowhead whale, eastern North Pacific, extralimital record

## Abstract

Bowhead whales occur in the Arctic year‐round. Their movements are largely correlated with seasonal expansions and reductions of sea ice, but a few recent extralimital sightings have occurred in the eastern and western North Atlantic and one was also documented in the western North Pacific over 50 years ago. Here we present details of a juvenile bowhead whale that was photographed and filmed from above and below the water while it was skim‐feeding in Caamaño Sound, BC, Canada on May 31, 2016. This sighting occurred over 2000 km southeast from the nearest known range for this species in the Bering Sea at a time that most bowhead whales in that region would have been migrating northeast. This sighting represents the first and only documentation of a bowhead whale in the eastern North Pacific to date.

## Graphical abstract

On May 31, 2016, a juvenile bowhead whale with heavy scarring on its body was photographed and filmed from above and below the water while skim‐feeding in Caamaño Sound, BC, Canada. This documentation occurred over 2000 km southeast from the nearest known range for this species in the Bering Sea at a time that most bowhead whales in that region would have been migrating northeast. This sighting represents the first and only documentation of a bowhead whale in the eastern North Pacific to date.

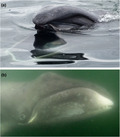

## 1 INTRODUCTION

The bowhead whale (*Balaena mysticetus*) has a circumpolar distribution in high‐latitude seas of the Northern Hemisphere and is adapted to living in close association with sea ice year‐round. Throughout its range, it preys on several species of zooplankton (Lowry et al., 2004) using a variety of techniques from skim‐feeding to benthic foraging (Würsig et al., 1985, 1989).

Four geographically defined stocks of bowhead whale are recognized (Baird & Bickham, 2021) and all were heavily depleted by commercial whaling (Thewissen & George, 2021). The Bering‐Chukchi‐Beaufort Seas (BCB) stock migrates north from Bering to Chukchi and Beaufort Seas in spring as sea ice recedes and returns in the autumn as sea ice expands (Braham et al., 1984). This population was estimated to number between 9000 and 14,000 individuals prior to commercial whaling (Brandon & Wade, 2006) but has been growing at a rate of 3.7% per year since 1978 and in 2011 had an estimated population size of over 16,000 individuals (Givens et al., 2016). By comparison, less is known of the Okhotsk Sea stock, but it is thought to be confined to the Okhotsk Sea (Shpak & Paramonov, 2018). It may have numbered over 6000 prior to whaling, but now likely consists of fewer than 400 individuals (Ivanshchenko & Clapham, 2010). Both the East Canada‐West Greenland (ECWG) and the East Greenland‐Svalbard‐Barents Sea (EGSB) stocks probably numbered in the tens of thousands prior to commercial whaling (Allen & Keay, 2006; Givens & Heide‐Jørgensen, 2021), but most recent population estimates for each are now around 6500 and 300, respectively (Doniol‐Valcroze et al., 2019; Hansen et al., 2018). Indigenous subsistence hunts for bowhead whales from the BCB and ECWG stocks continue in several Arctic communities (Suydam & George, 2021).

Most extralimital records of bowhead whales are in the North Atlantic where a few individuals have been documented in Newfoundland, the Gulf of Maine, England, Ireland, France, Belgium, and the Netherlands between 1998 and 2017 (Accardo et al., 2018; de Boer et al., 2017; Ledwell et al., 2007, 2014; Ledwell & Huntington, 2009; Whooley & Berrow, 2019). Additionally, in the 16^th^ century, bowhead whales were harvested in the Gulf of St Lawrence, which is much further south than their current range (Rastogi et al., 2004). The only documentation of a bowhead whale in the North Pacific that was outside the distribution range of the BCB or Okhotsk Sea stocks was a juvenile found and captured in Osaka Bay, Japan on June 23, 1969 (Nishiwaki & Kasuya, 1970).

## 2 METHODS AND RESULTS

**FIGURE 1:**
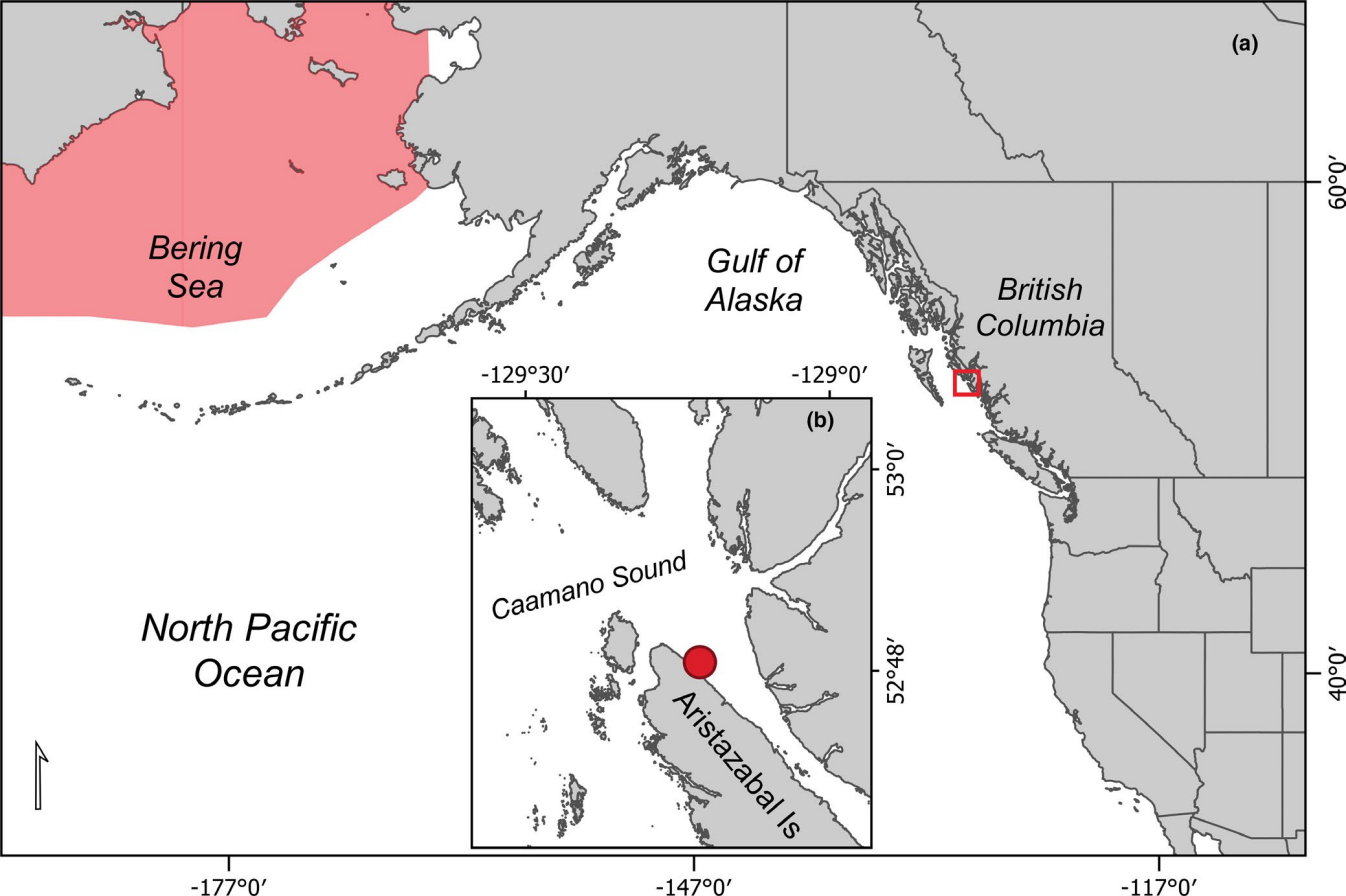
(a) Map of eastern North Pacific relative to typical bowhead whale distribution (light red shading) in the Bering Sea with red box and inset (b) showing the location of this bowhead whale sighting in Caamaño Sound, British Columbia, Canada. Bowhead whale range polygon credit: Cooke & Reeves, 2018

**FIGURE 2:**
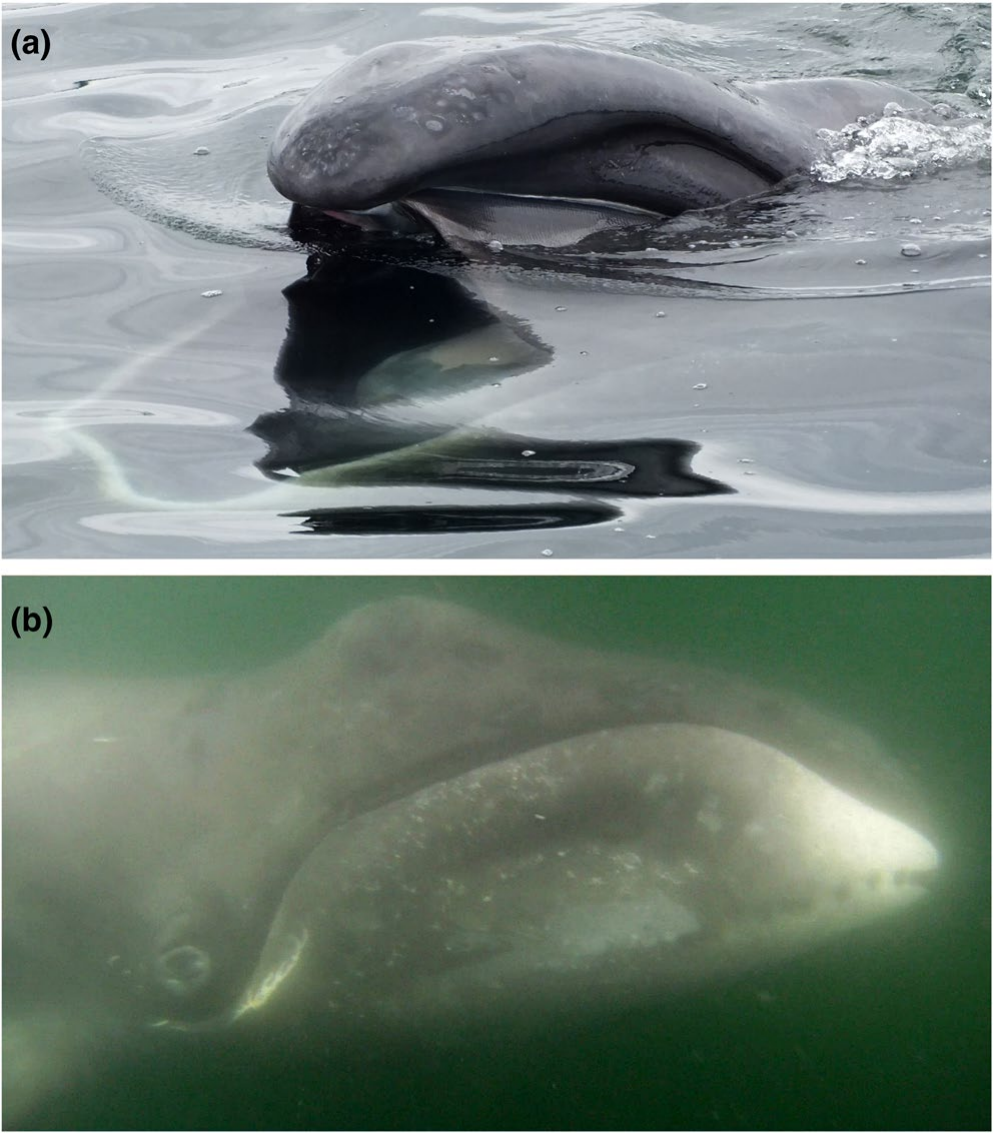
(a) Photos of the bowhead whale skim‐feeding at surface and (b) looking at underwater camera in Caamaño Sound, British Columbia, Canada on May 31, 2016

On May 31, 2016, a juvenile bowhead whale was documented in Caamaño Sound, British Columbia, Canada (Figure 1) within an area recognized as traditional territory by the Kitasoo/Xai'xais Nation and the Gitga'at Nation. This region includes a complex coastal network of inlets utilized by commercial and recreational vessel traffic. The sighting was made opportunistically by E.A.M and E.V.M at approximately 11:00 a.m. from a small aluminum boat off Baker Point on Aristazabal Island at 52˚48.5’ N–129˚12.8’ W. The whale was observed skim‐feeding at the surface (Figure 2a; Video 1) approximately 500 m from shore in about 50 m of water. It did not appear to react to the presence of the vessel when photographed and filmed both above and below the water from a minimum distance of about 1 m (Figure 2b). Several diagnostic features of this species were apparent including large head, arched mouthline, white patch with dark spots at the front of the lower jaw, and lack of dorsal fin (Figure 2a, b; Video 1). Its length was estimated to be similar to the vessel, that is, 7–8 m. Two somewhat parallel white scars approximately 2 m in length were visible along the anterior dorsal region and additional white scarring was visible around the leading edge of the flukes (Video 1). As many as 15–25 humpback whales (*Megaptera novaeangliae*) were also in the same general area as the bowhead whale. After about 30 min of documentation, the whale was left and, as far as we know, it was never seen again.

**VIDEO 1:**
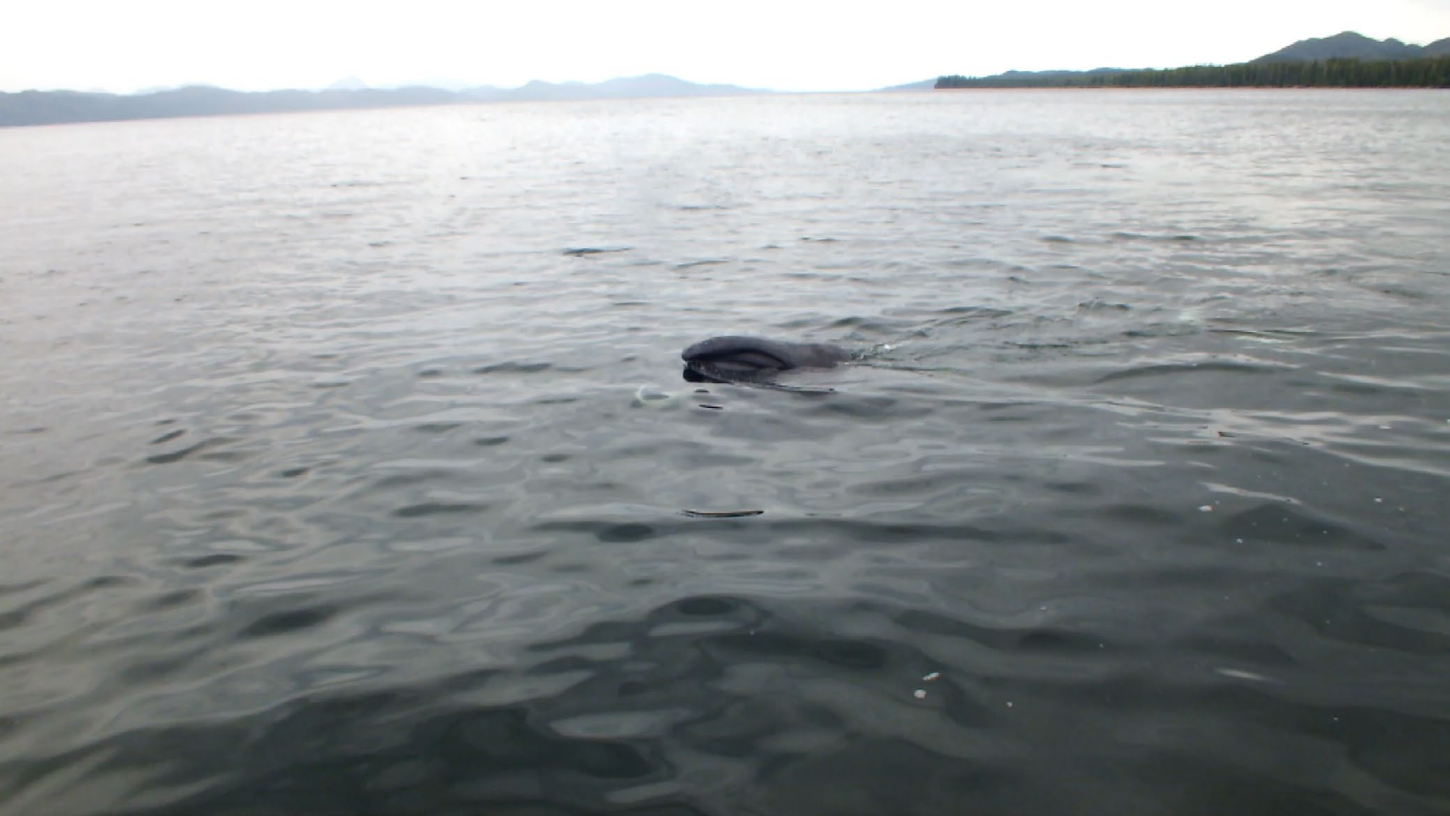
Video of juvenile bowhead whale with scars on anterior dorsal region and around leading edge of flukes skim‐feeding in Caamaño Sound, British Columbia, Canada on May 31, 2016

## 3 DISCUSSION

Changes in the occurrence and distribution of bowhead whales may over time be driven by expansions and reductions in sea ice coverage at high latitudes that enhance or restrict the habitat individuals are able to explore and exploit (Dyke et al., 1996; Foote et al., 2013; Heide‐Jørgensen et al., 2012; Laidre et al., 2008). It is expected that the range of some ice‐associated species like the bowhead whale will generally contract as ice continues to melt in the Arctic (Chambault et al., 2018; van Weelden et al., 2021). Yet, this loss of sea ice has enhanced primary production (Lewis et al., 2020), and although the sustainability of this relationship remains unclear (Tremblay & Gagnon, 2009), the related increase in prey availability for bowhead whales has improved body condition of individuals (George et al., 2015) and growth of the BCB population (Givens et al., 2016).

Extralimital records can be considered part of the natural dispersal process of growing populations. However, the bowhead whale reported here occurred over 2000 km southeast of the Bering Sea during a time that most individuals in the BCB population would have been migrating northeast toward the Chukchi and Beaufort Seas (George et al., 2004). This movement could be a case of overshooting on a mis‐orientated or reverse migration. This is a phenomenon well documented in birds (Howell et al., 2014) and may help define other extralimital occurrences of cetaceans such as an Antarctic minke whale (*Balaenoptera bonaerensis*) reported in the North Atlantic (Glover et al., 2010) and gray whales (*Eschrichtius robustus*) reported in the North and South Atlantic, respectively (Elwen & Gridley, 2013; Scheinin et al., 2011). The drivers of such movements are varied and largely unknown, but temporary yet significant disturbances to the Earth's magnetosphere caused by solar storms are known to disrupt navigation in cetaceans (Ferrari, 2017). They are hypothesized to have severely impacted the movements of several individuals in early 2016 (Vanselow et al., 2018) which is interesting given the timing of this sighting.

A commonality among a large proportion of extralimital encounters, including those involving bowhead whales, is that the animals involved were young (Accardo et al., 2018; de Boer et al., 2017; Ledwell et al., 2007; Nishiwaki & Kasuya, 1970). The length of the bowhead whale reported here is consistent with the lengths of young juveniles ≤5 years old (Koski et al., 2010; Lubetkin et al., 2012), and the scarring on its body may be evidence that this individual survived entanglement in fishing gear, attack by killer whales (*Orcinus orca*), or both (George et al., 2017; Reinhart et al., 2013). About 12% of bowhead whales harvested from the BCB population show evidence of line entanglement wounds (George et al., 2017), whereas killer whale attacks on bowhead whales are becoming more common as longer ice‐free periods provide more access for killer whales to hunt in Arctic waters (Clarke et al., 2013; George et al., 2017; Higdon et al., 2012). They tend to target younger individuals, as suggested by examination of carcasses (Suydam et al., 2016; Willoughby et al., 2020; Young et al., 2020) and higher rates of accumulation of scars caused by killer whale teeth on juveniles and sub‐adults than on adults (Reinhart et al., 2013).

Bowhead whales, like other cetacean species (Ford & Reeves, 2008; Laidre et al., 2006; Westdal et al., 2016), will shift their distribution toward shore or the ice edge to avoid killer whales (Matthews et al., 2020; Shpak & Paramonov, 2018). This young individual was found feeding at the surface in relatively close proximity to shore. However, its location was in one of few regions along the British Columbia coast where other baleen whales [humpback and fin whales (*Balaenoptera physalus*)] can regularly be found feeding on zooplankton near shore (Keen et al., 2017). This area is also where two rare sightings of endangered basking sharks (*Cetorhinus maximus*) occurred on July 17, 2017 and July 30, 2019, one of which was skim‐feeding only a few kilometers away from the location of the bowhead sighting (J. Pilkington *unpubl. data*).

The occurrence of a juvenile bowhead whale in Caamaño Sound, British Columbia represents the first confirmed record of this species in the eastern North Pacific despite intensive whaling efforts that took place in this region throughout the 19th and early 20th centuries (Clapham et al., 1997; Gregr et al., 2000; Reeves et al., 1985; Townsend, 1935) and significant cetacean research effort from Alaska to California in more recent decades (Barlow, 2016; Ford et al., 2010; Nichol et al., 2017; Rone et al., 2017; Wright et al., 2021). This sighting also represents the first natural occurrence in Pacific Canadian waters of any of the three cetacean species that are year‐round residents to the Arctic [bowhead whale, narwhal (*Monodon monoceros*), beluga (*Delphinapterus leucas*)], as most extralimital cetacean occurrences in western Canada are of tropical species associated with warm water events (Halpin et al., 2018).

## CONFLICT OF INTEREST

The authors declare they have no competing interests.

## AUTHOR CONTRIBUTIONS

**Jared R. Towers:** Conceptualization (equal); Investigation (equal); Resources (equal); Software (equal); Writing – original draft (lead); Writing – review & editing (lead). **James F. Pilkington:** Conceptualization (equal); Investigation (equal); Resources (equal); Software (equal); Writing – original draft (supporting); Writing – review & editing (supporting). **Ernest A. Mason:** Data curation (equal); Resources (equal); Writing – review & editing (supporting). **Ernest V. Mason:** Data curation (equal); Resources (equal); Writing – review & editing (supporting).

## Data Availability

All data collected during the sighting presented here are included in this published article and its supplementary video file.
